# SIMPLE/LITAF Expression Induces the Translocation of the Ubiquitin Ligase Itch towards the Lysosomal Compartments

**DOI:** 10.1371/journal.pone.0016873

**Published:** 2011-02-04

**Authors:** Heather E. Eaton, Guillaume Desrochers, Samuel B. Drory, Julie Metcalf, Annie Angers, Craig R. Brunetti

**Affiliations:** 1 Department of Biology, Trent University, Peterborough, Canada; 2 Département de Sciences Biologiques, Université de Montréal, Station ‘Centre-Ville’, Montréal, Canada; University of Florida, United States of America

## Abstract

LITAF is a small cellular protein with an unknown function. The C-terminus of LITAF contains a highly conserved domain termed the SIMPLE-like domain (SLD), while the N-terminus contains two PPXY motifs that mediate protein-protein interactions with WW-domain containing proteins. LITAF also harbors two endosome/lysosome targeting sequences at its C-terminus, but there has been conflicting reports regarding its intracellular localization. Here, we demonstrate that LITAF is localized to the late endosome/lysosomal compartment in a variety of cell lines. We also show that Itch, a WW-domain containing protein, and LITAF strongly interact and that this interaction depends on the two PPXY motifs in the N-terminus of LITAF. Interestingly, co-expression of LITAF with Itch induces major changes in Itch intracellular localization, bringing Itch from the trans-Golgi network to lysosomes. We show that this re-localization is dependent upon the interaction with the PPXY sequences of LITAF, since disruption of these binding motifs completely abrogates Itch re-localization.

## Introduction

LITAF (lipopolysaccharide-induced tumor necrosis factor-alpha factor), also known as SIMPLE (small integral membrane protein of the lysosome/late endosome) and PIG-7 (p53 inducible gene-7) was first identified as a gene that was up-regulated in response to bacterial cell wall components, including lipopolysaccharide (LPS), and was therefore proposed to be a pathogen-associated molecular pattern (PAMP)-inducible gene [1], [2], [3]. LITAF is predicted to encode a 161 amino acid protein. The N-terminus of LITAF contains two PPXY (PY) motifs responsible for binding to WW domain oxidoreductase (WWOX), neuronal precursor cell expressed developmentally downregulated 4 (Nedd4), and tumor suppressor gene 101 (TSG101) [4], [5], [6]. The C-terminus of LITAF is 68 amino acids long and contains a modified RING-finger domain composed of a CX_2_C motif, a long (approximately 25 amino acid) hydrophobic region, and a HXCX_2_C motif. This interrupted RING-finger has been termed the SIMPLE-like domain (SLD) [1]. This domain is found in a wide range of species (including yeast, plants, insects, and humans) and represents a new family of proteins with unknown function [1]. Other functions that have been ascribed to LITAF [2], [7], [8], [9] have been called into question with evidence from a number of groups suggesting that the LITAF used in these experiments contained a nucleotide insertion that altered the reading frame in the C-terminal half of the protein, thereby eliminating the SLD [1], [10], [11].

In addition to the SLD, the C-terminus of LITAF contains the carboxyl terminus lysosomal targeting sequence YXXΦ (where Φ is any bulky hydrophobic amino acid) [1]. Currently, the localization of LITAF remains unclear and may be cell type specific [6]. LITAF has been found to localize to late endosomes/lysosomes [1], the Golgi apparatus [5], [6], and to the plasma membrane [6]. Although cellular localization of LITAF is inconsistent, it does appear that LITAF localizes along secretory and lysosomal degradation pathways.

There are several lines of evidence that suggest that LITAF may function in protein degradation. First, E3 ubiquitin ligases, which are involved in ubiquitin-mediated degradation of proteins, often contain RING-finger domains [6], [12], [13]. LITAF contains a modified RING-finger domain. However, it is not clear how the hydrophobic amino acids present within the RING-finger domain of LITAF affect its function. Second, LITAF mutations are associated with Charcot-Marie-Tooth (CMT) disease [10], [14], [15]. CMT is an inherited peripheral neuropathy that can be characterized by protein aggregates [16], suggesting a putative role for LITAF in protein degradation. Last, binding partners of LITAF, including Nedd4 and TSG101, are involved in lysosomal degradation of proteins. Nedd4 is a member of a family of HECT containing E3 ubiquitin ligases. This family of proteins shares a common structure, which includes an N-terminal C2 domain, 2–4 WW domains, as well as a C-terminal HECT domain. Nedd4 acts at the plasma membrane and the Golgi apparatus to mono-ubiquitinate substrates for degradation in the lysosome [17]. TSG101, another binding partner of LITAF [6], operates downstream of Nedd4. TSG101 acts to recognize and sort mono-ubiquitinated substrates into multivesicular bodies for future lysosomal degradation [18], [19].

The interaction between LITAF and Nedd4 or WWOX is mediated by PPXY motifs found in the N-terminus of LITAF [4]. Itch is another member of the Nedd4/Nedd4-like HECT E3 family that binds to PPXY motifs via its WW domains. Itch, a homologue of the human atrophin-1-interacting protein 4 (AIP4), was first identified as a gene disrupted in non-agouti-lethal 18H mice that develop a spectrum of immunological diseases and constant itching of the skin [20]. The Itch gene encodes an 864 amino acid protein that regulates important cellular functions by inducing proteasomal degradation of a variety of substrates. As it is demonstrated by the a^18H^ phenotype, Itch plays a role in the immune response by binding c-jun and JunB via its WW domains. Itch induces ubiquitination and degradation of these transcription factors involved in T_H_2 differentiation, providing a molecular link between Itch deficiency and the itching phenotype [21]. Itch's WW domains also bind to a PPPY motif in the C-terminus of p73, inducing its ubiquitination and degradation. This transcription factor is involved in the response to DNA damage and in cell cycle control, providing another role for the ligase [22]. Furthermore, the implications of Itch also extend to cell death by promoting c-FLIP turnover, an anti-apoptotic protein inhibiting caspase-8 [23]. Itch also acts as a key molecule between EGF signaling and cell survival through downregulation of tBid, an important intermediate in ligand-induced apoptosis via caspase-3 activation [24]. Itch does not only affect receptor signaling, but can also influence EGFR stability at the plasma membrane by controlling the expression of Cbl and Endophilin, two trafficking proteins required for receptor endocytosis [25].

The Itch ligase localizes to the trans-Golgi network and to endosomal compartments, which confers the capacity to interact with internalized proteins and their endocytic accessory proteins and cause their proteasomal degradation, which affects protein trafficking [26]. Similar to Itch, LITAF has also been reported to localize to late endosomes [1], raising the possibility that these proteins may interact *in vivo* and influence each other's activity.

Here, we report that Itch strongly interacts with LITAF, and that this interaction relies on the WW domains of Itch and on the two PPXY motifs found in the N-terminus of LITAF. Interestingly, co-expression of LITAF with Itch induces major changes in Itch intracellular localization, bringing Itch to the lysosome. We show that this re-localization is dependent upon the interaction with LITAF, since disruption of the binding motifs completely abrogates Itch re-localization. In contrast, although Nedd4 also interacts with LITAF, it is not re-localized upon expression of LITAF.

## Results

### LITAF interacts with Itch

**Figure 1:**
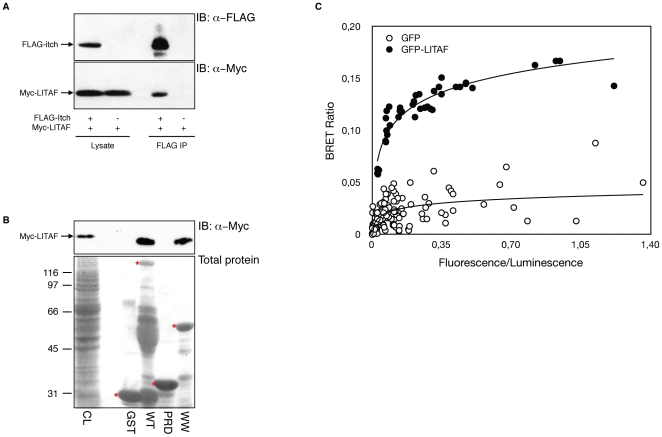
LITAF interacts with Itch *in vitro* and *in vivo*. (A) HEK-293T cells were transiently transfected with FLAG-Itch with or without co-transfection of myc-LITAF. Total cell lysates were blotted with anti-FLAG and anti-myc to show protein expression (lower panel), and immunoprecipitated with anti-FLAG to reveal LITAF co-immunoprecipitation (upper panel). (B) Extracts from 293T cells transfected with myc-LITAF were incubated with either GST alone, GST-Itch WT, GST-Itch PRD or GST-Itch WW pre-coupled to glutathione-Sepharose. Input proteins is shown in the first lane (CL). Proteins bound to GST beads are shown in the next lanes. Immunoblotting with anti- myc antibodies shows the presence of myc-LITAF (upper panel). Total gel loading is shown by ponceau staining of the blot to reveal GST loading. The bands representing the GST-fusions in the Ponceau staining are marked by a red asterisk. Additional staining in the GST-Itch-WT lane likely represents degradation products of the fusion protein. (C) 293T cells were co-transfected with constant amount of rLuc-Itch and various amounts of either GFP alone or GFP-LITAF. The graph is a representative example of the saturation studies performed to provide evidence for a specific interaction between the proteins. BRET ratios were plotted as a function of the excited GFP activity to total rLuc activity ratio, allowing comparison of BRET ratios between the negative control GFP and GFP-LITAF when expressed at the same level.

LITAF is known to interact via its PY motifs with the WW domains of Nedd4, an ubiquitin ligase of the C2-WW-HECT family, and this interaction occurs at the plasma membrane and Golgi apparatus [4], [6]. All ligases of this family have highly homologous WW domains and are able to bind PY motifs. Of these, Itch presents an intracellular localization with enrichment in Golgi/endosomal compartments, displaying potential overlapping localization with LITAF [1], [26], [27]. Therefore, one could expect that LITAF and Itch will interact. To verify this hypothesis, we transfected HEK-293T cells with myc-LITAF in the presence or absence of FLAG-Itch. We then immunoprecipitated transfected cell extracts with a monoclonal antibody against FLAG and looked for the presence of myc fusion proteins in the immunoprecipitated fractions. When FLAG-Itch was immunoprecipitated we were able to detect myc-LITAF by Western blot, demonstrating the interaction between Itch and LITAF (Figure 1A). We then refined these results using pull-down assays to determine which domain of Itch is involved in the binding. Wild-type myc-LITAF expressed in HEK-293T cells specifically bound to GST-fusion proteins of full-length Itch and the isolated WW domains of Itch, but failed to interact with GST alone or the GST-fused PRD domain of Itch. This experiment confirmed that the WW domains of Itch are sufficient to mediate the interaction with LITAF (Figure 1B). GST-fusion proteins are shown in the Ponceau staining below the immunoblot. Note that the GST-Itch-WT fusion is showing several degradation bands, as typically seen with this particular fusion protein (e.g., [28]).

To determine if the interaction occurred in living cells, we used bioluminescent resonance energy transfer (BRET) using HEK-293T cells co-transfected with Itch fused to Renilla luciferase (rLuc-Itch) and GFP-LITAF. Coelenterazine degradation by rLuc generates non-radiative resonance energy that is transferred from the emitting rLuc to GFP, which becomes excited and in turn emits fluorescence when rLuc and GFP are in close proximity (≤100 A°) as a consequence of fusion protein interaction. A BRET ratio is calculated for each transfection condition, as detailed in Materials and Methods. A significant BRET signal was measured only in cells co-transfected with rLuc-Itch and GFP-LITAF, whereas only a background-level signal was generated by cells co-transfected with rLuc-Itch and GFP (Figure 1C). This figure shows a representative example of an increasing BRET ratio with increased GFP fusion expression, whereas rLuc was kept relatively constant. Similar results were obtained with rLuc-LITAF and GFP-Itch (not shown). These results together confirm that LITAF and Itch interact in living cells.

### LITAF localizes to late endosomes/lysosomes

**Figure 2:**
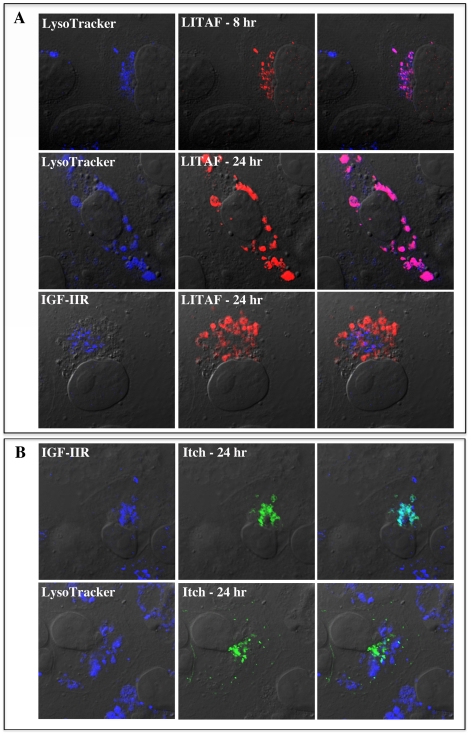
LITAF and Itch cellular localization. BGMK cells were transiently transfected for 8 or 24 hours with (A) FLAG-LITAF or (B) GFP-Itch. Live cells were initially incubated with LysoTracker followed by fixation and permeabilization. Cells then underwent indirect immunofluorescence using anti-IGF-IIR (trans Golgi-network; blue) and/or anti-FLAG antibodies (LITAF; red). GFP-Itch is shown in green. Differential interference contrast (DIC) was used to visualize cells and images were captured using a laser scanning confocal microscope.

We then confirmed the subcellular localization of LITAF, since there is discrepancy in the literature about its localization and it is suggested to be cell type specific [6]. Due to the fact that endogenous LITAF levels are below detection, we transiently transfected FLAG-LITAF or myc-LITAF into BGMK cells at different time points. At all time points we observed strong vesicular staining throughout the cytoplasm, which localized with the late endosome/lysosome marker LysoTracker, but not with the trans-Golgi network marker IGF-IIR (Figure 2A). Similar results were seen in HEK-293T, Cos-7 cells, and PAE cells (Supporting figure S1). This demonstrates that LITAF localizes to late endosomes/lysosomes in several cell lines.

### Subcellular localization of Itch is altered by the presence of LITAF

**Figure 3:**
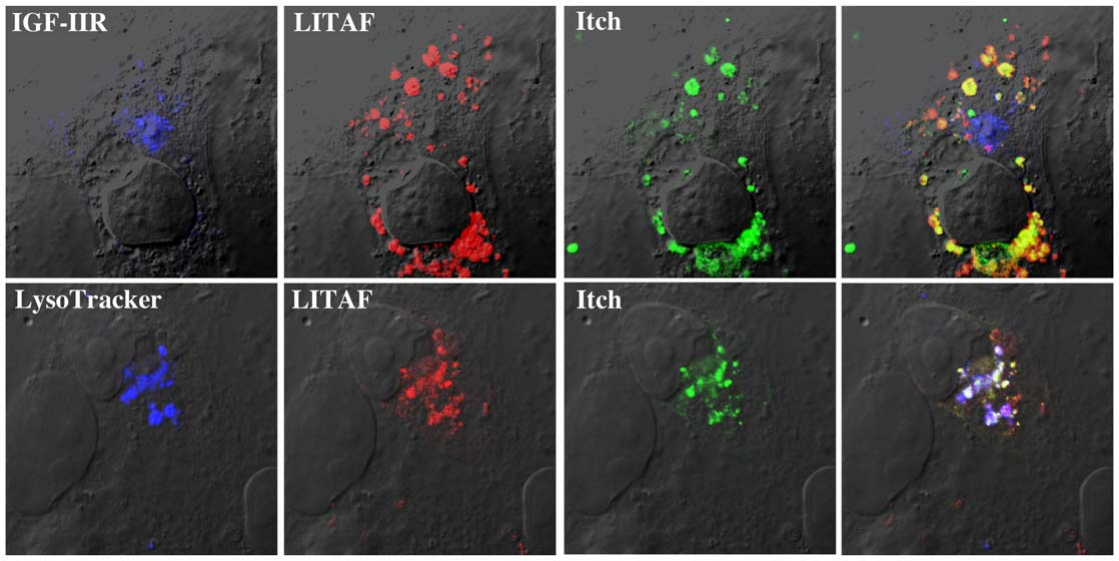
LITAF changes the cellular localization of Itch. FLAG-LITAF was transiently co-transfected into BGMK cells with GFP-Itch. Sixteen hours post-transfection, cells were probed with LysoTracker (blue) and fixed. LITAF was detected using anti-FLAG antibodies (red) while the trans-Golgi network was identified using anti-IGF-IIR antibodies (blue). GFP-Itch is shown in green. Cells were visualized using DIC.

In order to determine the extent of Itch and LITAF overlap in their subcellular localization, we first examined Itch localization in BGMK and HEK-293T cells. FLAG or GFP-Itch was transfected into cells that were then probed with LysoTracker or IGF-IIR. As previously demonstrated [26], we found extensive co-localization between Itch and a marker of the trans-Golgi network (IGF-IIR), but no co-localization with LysoTracker, a late endosome/lysosome marker (Figure 2B). We saw no overlap between GFP-Itch and LysoTracker, as well as between FLAG-LITAF and IGF-IIR (Figure 2A and B), suggesting that both proteins do not reside in the same compartment. Surprisingly, when FLAG-LITAF was transiently co-transfected along with GFP-Itch, LITAF and Itch were found to co-localize within the cell along with LysoTracker and not with IGF-IIR (Figure 3). This data suggests that LITAF is able to alter the cellular localization of Itch from the trans-Golgi network to the lysosomes.

### Two PPXY motifs in LITAF are required for the interaction between Itch and LITAF

**Figure 4:**
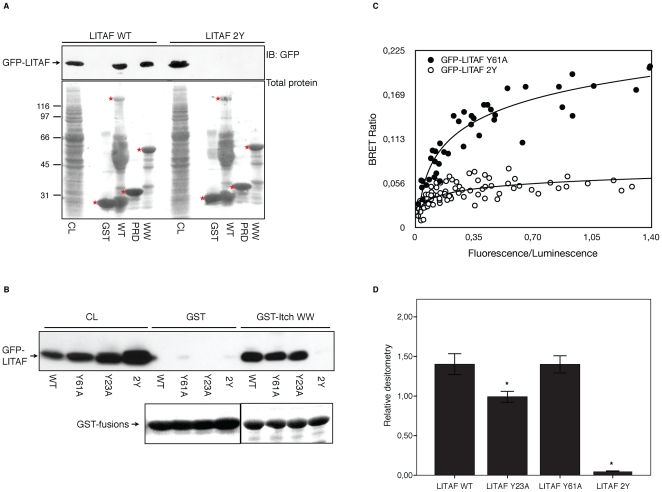
Mutation of both PPXY domains disrupts Itch and LITAF interaction. (A) Extracts from HEK-293T cells transfected with GFP-LITAF WT or GFP-LITAF Y23,61A were incubated with either GST alone, GST-Itch WT, GST-Itch PRD or GST-Itch WW pre-coupled to glutathione-Sepharose. Aliquot from total cell lysate (CL) and proteins specifically bound to the beads were processed by immunoblot with a polyclonal antibody against GFP. (B) HEK-293T cells were transiently transfected with either GFP-LITAF WT, GFP-LITAF Y61A, GFP-LITAF Y23A or GFP-LITAF Y23,61A. Aliquots of CL were processed by immunoblot with GFP antibody to show protein expression. The rest of the extracts were incubated with either GST or GST-Itch WW fusion proteins pre-coupled to gluthatione-Sepharose. Proteins specifically bound to the beads were immunoblotted with GFP antibody to reveal protein interactions. The bands representing the GST-fusions in the Ponceau staining are marked by a red asterisk. Additional staining in the GST-Itch-WT lane likely represents degradation products of the fusion protein. (C) 293T cells were co-transfected with constant amount of rLuc-Itch and various amounts of either GFP-LITAF Y61A or GFP-LITAF Y23,61A. The graph is a representative example of the saturation studies performed to provide evidence for a specific interaction between the proteins. BRET ratios were plotted as a function of the excited GFP activity to total rLuc activity ratio, allowing comparison of BRET ratios between GFP-LITAF Y61A and GFP-LITAF Y23,61A when expressed at the same level. (D) Quantification of the interaction between Itch WW domains and the different LITAF constructs. The densitometry of GFP signal in the fraction bound to GST-Itch-WW beads relative to the densitometry of the GFP signal in 1/10 volume of protein extract is represented as described in materials and methods. Data are mean ± s.e.m. from n = 4 experiments. * p<0.05 compared to binding of GFP-LITAF WT (ANOVA post-hoc Tukey).

Interactions between Itch and substrate proteins occur via Itch's WW domains. The WW domains recognize and bind to proline rich PY domains, including PPXY motifs. LITAF contains two PPXY motifs within its N-terminus and while Itch is suspected to bind to LITAF through these PPXY elements, it is unclear whether a single PPXY motif or both PPXY motifs are required for binding. Using site-directed mutagenesis the PPXY motifs of LITAF were mutated to PPXA, which abolishes binding to WW domains [29]. Three N-terminal GFP-tagged LITAF constructs were generated. We then conducted pull-down assays using these constructs and GST-Itch to determine the interaction between Itch and the mutated forms of LITAF. When both PPXY sites were mutated, LITAF did not interact with any Itch domain, demonstrating the WW domain binding to PPXY motifs is the only interaction site between the two proteins (Figure 4A). However, LITAF-WT and both of the single PY mutants expressed in HEK-293T cells strongly bound to the WW domains of Itch fused to GST (Figure 4B). The double mutant alone was unable to bind the GST-fused WW domains of Itch (Figure 4B). This experiment shows that the mutation of a single PY motif of LITAF is not sufficient to disrupt the interaction with Itch. On the other hand, the mutation of both PY motifs completely abolished the interaction (Figure 4B). Similar results were obtained in living cells using BRET, where disruption of a single PPXY motif mutation did not significantly alter the binding of LITAF to Itch, but the double mutant completely lost all BRET signal (Figure 4C). To determine if both binding sites contributed equally, we performed a quantitative experiment to compare binding of the different forms of LITAF to Itch WW domains. Densitometry analysis of four identical experiments show that while mutating LITAF-Y23A slightly decreased binding, the mutation of LITAF-Y61A had no significant effect compared to WT. As demonstrated before, mutation of both tyrosines completely abolished Itch binding (Figure 4D). These results show that Itch WW domains can recognize both PY motifs with a small preference towards the first PPXY motif. However, the loss of a single binding site is not sufficient to prevent binding.

### Itch re-localization to lysosomes is mediated by an interaction with LITAF

**Figure 5:**
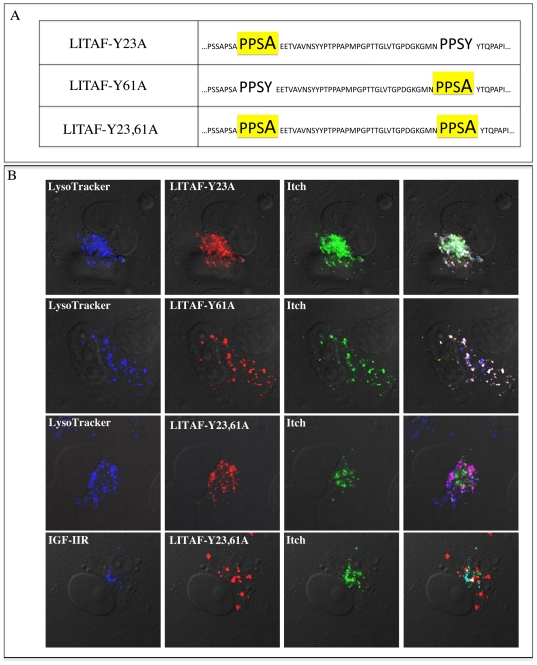
Mutation of both PPXY domains disrupts Itch and LITAF co-localization. (A) Site-directed mutagenesis was used to mutate the two PPXY motifs (either individually or together) located in the N-terminus of GFP-LITAF. (B) Expression constructs containing each mutated GFP-LITAF construct and FLAG-Itch were co-transfected into BGMK cells and 16 hours post-transfection, cells were incubated with LysoTracker (blue), fixed, and processed by indirect immunofluorescence to detect Itch (anti-FLAG; green), LITAF (red), and the trans-Golgi network (anti-IGF-IIR; blue). Cells were visualized using DIC.

Since both LITAF PPXY motifs are able to interact with Itch, we next tested the LITAF mutants for their ability to alter the localization of Itch (Figure 5A). When GFP-LITAF-Y23A was transiently co-transfected into cells with FLAG-Itch, it was found that some co-localization remained between LITAF and Itch, and that staining overlapped with LysoTracker (Figure 5B). Transient transfection of GFP-LITAF-Y61A along with FLAG-Itch showed high levels of co-localization between LITAF and Itch (Figure 5B). The overlap between LITAF-Y61A and Itch showed co-localization with LysoTracker (Figure 5B). When the LITAF double mutant (GFP-LITAF-Y23,61A) was transfected into BGMK cells along with FLAG-Itch, no overlap was found between LITAF Y23,61A and Itch (Figure 5B). LITAF-Y23,61A localized along with LysoTracker suggesting that mutation of either PPXY motif has no effect on the subcellular localization of LITAF (Figure 5B). While LITAF-Y23,61A remained localized to the lysosome, Itch remained co-localized with the marker IGF-IIR suggesting that the interaction between Itch and LITAF is critical for the re-localization of Itch upon LITAF expression. Disruption of LITAF PPXY motifs abolished the interaction with Itch.

We next sought to determine if the endogenous Itch protein was equally susceptible to LITAF-induced relocalization as overexpressed FLAG-Itch. We used COS-7 cells, HEK-293-T cells and PAE cells grown on glass coverslips and transfected with either GFP-LITAF-WT or GFP-LITAF-Y23,61A. In non-transfected cells, anti-Itch staining is visible throughout the cytoplasm, with concentration in areas near the trans-Golgi network and endosomal compartments. Endogenous Itch localization, although more diffused, is similar to overexpressed FLAG or GFP-Itch. In cells expressing GFP-LITAF-WT, we observe a significant accumulation of Itch in GFP-LITAF-WT-rich puncta. Colocalization of Itch and LITAF-WT can be observed in all examined cell lines (Supporting figure S1). In contrast, overexpression of GFP-LITAF-Y23,61,A does not alter Itch distribution as compared to non-transfected cells, or cell transfected with GFP only (Supporting figure S1).

### LITAF is unable to alter the cellular localization of Nedd4

**Figure 6:**
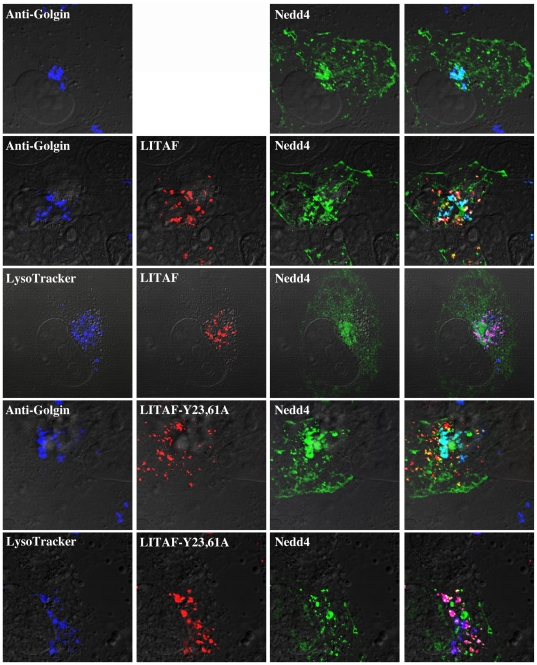
LITAF does not alter the cellular localization of Nedd4. YFP-Nedd4 was transiently transfected into BGMK cells either alone, with myc-LITAF WT, or myc-LITAF Y23,61A. Cells were fixed and immunofluorescence was completed to visualize WT LITAF or LITAF Y23,61A (anti-myc: red), Nedd4 (green), lysosomes (LysoTracker; blue) and the Golgi apparatus (anti-Golgin; blue). Cells were visualized using DIC.

Since LITAF causes the relocation of Itch, we wanted to determine if LITAF caused relocation of other WW domain containing proteins. Nedd4 is a WW domain containing E3 ubiquitin ligase that has previously been shown to interact with LITAF [4], [6]. YFP-Nedd4 was transiently transfected and the Nedd4 protein was found localized to the perinuclear region, the plasma membrane, and also exhibited some cytoplasmic vesicular staining (Figure 6). Staining in the perinuclear region of the cell co-localized strongly with the Golgi marker, Golgin 97 (Figure 6). Co-transfection of myc-LITAF and YFP-Nedd4 into BGMK cells resulted in some, but not complete, co-localization between the two proteins (Figure 6). While some overlap was present, LITAF remained localized to the late endosomes/lysosomes while Nedd4 localization remained consistent with the expression pattern when Nedd4 was transfected alone (Figure 6). To determine if the PPXY motifs of LITAF affected Nedd4 localization, YFP-Nedd4 was also transiently co-transfected with the LITAF double mutant (myc-LITAF Y23A,61A). There was no change in localization of Nedd4 when co-transfected with the double mutant (Figure 6). Nedd4 remained localized to the Golgi with a small amount of localization with LITAF/lysosomes. This highlights that the behavior of Nedd4 is different from that of Itch in the presence of LITAF. This suggests that the ability of LITAF to interact with and alter the cellular localization of Itch is unique and not a common feature to other WW domain containing proteins.

## Discussion

Here we describe a novel interaction between LITAF and the ubiquitin ligase Itch. This interaction resulted in a change of cellular localization of Itch from the trans-Golgi network to lysosomes, where it co-localized with LITAF. The interaction is specific and cellular re-localization was mediated through Itch's WW domains and the two PPXY motifs found in the N-terminus of LITAF.

The function of LITAF is currently unknown, although many pieces of evidence, including the ability of LITAF to interact with Nedd4 and TSG101, point to a role in the ubiquitin-mediated lysosomal degradation pathway [4], [6]. Nedd4 is an E3 ubiquitin ligase that contains several WW domains that interact with PPXY containing proteins and catalyzes ubiquitination through a catalytically active HECT domain. Ubiquitinated proteins then interact with TSG101, a vascular protein sorting (Vps) protein that binds to and sorts ubiquitinated proteins at the endosomal membrane [18], [19]. The interaction between TSG101 and substrate proteins is mediated through a proline rich P(S/T)AP motif.

In this study we demonstrated that LITAF not only binds to Nedd4 and TSG101, but also the E3 ubiquitin ligase Itch. Independently, Itch and LITAF localize to different compartments within the cell, specifically Itch localizes to the trans-Golgi network and LITAF localizes to late endosomes/lysosomes. Although endogenous LITAF could not be detected in our cell lines, overexpressed LITAF localized to lysosomes at 8 hours post-transfection suggesting that even at low levels LITAF is localized to lysosomes. This fact, along with previous localization of LITAF to the lysosome and the presence of a lysosomal targeting sequence in the C-terminus of LITAF suggests that the lysosomal localization of overexpressed LITAF is reliable [1]. In order for Itch and LITAF to interact they must localize, at least transiently, within the same compartment of the cell. The ubiquitin-mediated lysosomal degradation pathway is very dynamic and the trans-Golgi network, endosomes (early and late), and lysosomes are intricately linked with proteins shuttling rapidly from one location to another. It is highly likely then that Itch and LITAF are at least transiently within the same cellular compartment. However, LITAF must have a dominant sorting sequence to “pull” Itch out of the trans-Golgi network and into the late endosome/lysosome compartment. Interestingly, the interaction between LITAF and Itch may suggest a potential orientation for LITAF. Little is known about the orientation of LITAF in vesicles. The PPXY motifs of LITAF must be in the same compartment as the WW domains of Itch so the two proteins can interact *in vivo*. Since Itch is suspected to be a cytosolic protein associated with internal membranes, we deduce that the N-terminus of LITAF, containing the PPXY motif, must also be found in the cytosol. If the hydrophobic stretch of amino acids found in the SLD of LITAF act as a transmembrane domain than the C-terminus of LITAF may be found on the luminal side of endosomes/lysosomes. Future studies will further explore the orientation of LITAF in vesicle membranes.

Itch and Nedd4 are structurally similar proteins that are members of a conserved family of HECT ubiquitin ligases. Both contain an N-terminal C2 domain that may play a role in membrane targeting. They both contain 4 WW domains that mediate interactions with proline rich motifs along with a C-terminus HECT domain responsible for E3 ligase activity. Furthermore, both Itch and Nedd4 interact with LITAF, at least primarily through LITAF's first PPXY motif. There are several possible explanations as to why LITAF can mediate the re-localization of Itch, but not Nedd4. First, given the high levels of structural similarities between Itch and Nedd4, it suggests that functional differences between Nedd4 and Itch are responsible for the different situations induced following an interaction with LITAF. Another possibility is that the targeting sequences of Nedd4 are “stronger” than the targeting sequences for Itch. This would imply that although LITAF and Nedd4 can interact, LITAF is not able to mediate the re-localization of Nedd4. Finally, *in vivo*, LITAF and Nedd4 may not be present in the same cellular compartments. If the two proteins cannot physically interact *in vivo*, then there is no possibility of LITAF mediating the re-localization of Nedd4. Our immunofluorescence data suggests that Nedd4 is found in the Golgi apparatus, but not in the trans-Golgi network. This may move Nedd4 out of the cycling pathway between the trans Golgi network/endosome/lysosome compartments precluding it from interacting with LITAF.

Since the function of LITAF remains unknown, we can only speculate on the consequences that the re-localization of Itch has on Itch and LITAF function. Itch may be sorted from the trans-Golgi network to the lysosomes with the assistance of LITAF. LITAF and Itch may form a stable complex that translocates to the lysosome where Itch may or may not dissociate from LITAF within the lysosome for future degradation. It is also possible that LITAF retains Itch in the late endosomes/lysosomes. Itch has been found to localize within both endosomes and the trans-Golgi network [26]. The presence of LITAF may limit movement of Itch and retain Itch within the late endosomes. LITAF may sequester Itch to limit the ability of Itch to target proteins for degradation or to protect the cell from the putative harmful effects of Itch through its degradation. Interestingly, ubiquitination of Jun by Itch has been shown to trigger Jun accumulation to the lysosomal compartment, by a still unknown mechanism [27]. The LITAF and Itch interaction could thus be a targeting mechanism to bring Itch substrates to the lysosome.

Binding of small PPXY motif-containing proteins to Itch may also impact its activity. Ndfip1 and Ndfip2 binding have been shown to stimulate the ubiquitin ligase activity of both Itch and Nedd4 [30], [31]. Conversely, N4BP1 strongly inhibits Itch-catalyzed polyubiquitination of several proteins by preventing the interaction between the ligase and its substrates, thereby reducing the transfer of ubiquitin molecules to Itch protein targets [32].

On the other hand, due to the fact that Itch is an E3 ubiquitin ligase, the possibility exists that Itch re-localizes to or remains in late endosomes where it interacts with LITAF and mediates the transfer of ubiquitin to LITAF for future degradation. LITAF may represent another substrate of Itch and Itch may function to regulate cellular levels of LITAF by targeting it for degradation in the lysosome. Further studies will be important to elucidate the consequences of the novel interaction between LITAF and Itch.

## Materials and Methods

### Reagents, cell lines, and antibodies

Baby green monkey kidney (BGMK) cells were obtained from the American Type Culture Collection (ATCC; Manassas, VA) and were maintained at 37°C with 5% CO_2_ in Dulbecco's modified Eagle's medium (DMEM; HyClone, Ottawa, ON) supplemented with 7% FBS, 2 mM L-glutamine, penicillin (100 U/mL), and streptomycin (100 µg/mL). Human embryonic kidney (HEK)-293T cells for immunoprecipitation, pull-down assays and BRET experiments were obtained from the ATCC and were maintained at 37°C with 5% CO_2_ in high glucose DMEM (Gibco products, Invitrogen, Grand Island, NY) supplemented with 10% cosmic calf serum (HyClone, Ottawa, ON), penicillin (Invitrogen, Burlington, ON; 100 U/mL) and streptomycin (Invitrogen, Burlington, ON; 100 µg/mL). All cells used for immunoprecipitation and pull-down assays were transfected with the indicated plasmids using calcium/phosphate [33] and 5 µg plasmid/10 cm^2^ plate. Cells used for immunofluorescence were transfected using a polyethylenimine (PEI) reagent using 5 µg plasmid/10 cm^2^ plate and a PEI:DNA ratio of 4∶1. Antibodies used during immunofluorescence include: 9E10 myc monoclonal antibody obtained from Roche (dilution - 1/100; Indianapolis, IN); monoclonal antibody against FLAG (M2) from Sigma (dilution - 1/500; Oakville, ON); anti-IGF-IIR from Santa Cruz Biotechnology (dilution – 1/50; Santa Cruz, CA); FITC/Cy3/Cy5-conjugated goat anti-mouse or anti-rabbit immunoglobulin G (IgG) from Jackson ImmunoResearch Inc. (dilutions – 1/100, 1/200, 1/100 respectively; West Grove, PA); anti-Golgin 97 antibody from Invitrogen (dilution – 1/100; Burlington, ON); and LysoTracker™DND-99 from Molecular Probes (Burlington, ON). Antibodies used during immunoprecipitations and pull-down assays include: monoclonal antibody against c-myc clone 9E10 obtained from Enzo Life Sciences (dilution 1∶1000; Farmingdale, NY); monoclonal antibody against FLAG (M2) from Sigma (dilution - 1/1000); and polyclonal anti-GFP from Invitrogen (dilution 1/5000).

### Expression plasmids

LITAF (wild-type; WT) expression plasmids containing N-terminus FLAG or myc tags were generated by PCR and cloned into the *Xho*I and *Hind*III restriction sites of the plasmid pcDNA3.1-A (Invitrogen) using mouse LITAF cDNA (MGC-6569, ATCC) as template DNA and the following primers: 5′-AAGCTTA TGGATTACAAGGATGACGACGATAAGTCGGTTCCAGGACCTTACC-3′ (F – FLAG), 5′-AAGCTTATGGAACAAAAAGTTATTTCTGAAGAAGATCTGTCGGTTCCAGGAC CTTACC-3′ (F–myc), 5′-CTCGAGCTAAAAGCGTTGTAGGTG-3′ (R). An LITAF WT expression plasmid containing N-terminus GFP tag was generated by PCR and cloned into the *Xho*I and *Hind*III restriction sites of the plasmid pEGFP-C2 (Clontech, Mountain view, CA) using myc-LITAF as template DNA and the following primers: forward 5′-GAGACTCGAGAATGTCGGTTCCAGGACC-3′ and reverse 5′-GAGAAAGCTTCTACAAACGCTTGTAGGTG-3′. The resulting GFP-LITAF plasmid was used as a template to make the 3 LITAF mutants Y23A, Y61A or Y23,61A using a QuikChange® Lightning Multi Site-Directed Mutagenesis Kit (Stratagene, La Jolla, CA) according to the manufacturer's instructions and with the following primers: Y23A 5′-CCACCCCCAACCGCTGAAGAAACAGTG-3′ and Y61A 5′-GAATCCACCTTCGG CCTACACCCAGCC-3′. A myc tagged N-terminal LITAF Y23,61A construct was created through amplification of LITAF Y23,61A from the GFP-LITAF Y23,61A template DNA using 5′-AAGCTTATGGAACAAAAAGTTATTTCTGAAGAAGAT CTGTCGGTTCCAGGACCTTACC-3′ as the forward primer and 5′-CTCGAGCTA AAAGCGTTGTAGGTG-3; as the reverse primer. It was cloned into pcDNA3.1-A (Invitrogen) using the restriction sites *Xho*I and *Hind*III. FLAG (pFlag-CMV2) or GST (pGEX-4T-1) tagged Itch (WT), Itch's PRD domains or WW domains have been described previously [26], [28]. Itch sequence was amplified by PCR from FLAG-Itch WT using forward 5′GAGAGGTAC CAATGGGTAGCCTCACCATG-3′and reverse 5′- GAGAGGATCCTTACTCTTGTC CAAATCCTTC-3′. The resulting PCR product was subcloned into the BamHI and KpnI restriction sites of the plasmid pRluc-C1 (BioSignal Packard, Montreal, QC). YFP-Nedd4 was a kind gift from Paul D. Bieniasz (Aaron Diamond AIDS Research Center and The Rockefeller University, New York, USA).

### Immunoprecipitation and pull-down assays

Dishes (10 cm) of transfected HEK-293T cells were washed in phosphate buffer saline (PBS) and resuspended in 1 mL buffer A (20 mM Hepes, pH 7.4, 150 mM NaCl) plus protease inhibitors. The cells were lysed by sonication and Triton X-100 was added to a final concentration of 1%. Extracts were incubated for 20 minutes at 4°C and centrifuged at 45 000 rpm in an ultracentrifuge at 4°C. For immunoprecipitation assays, extracts of transfected cells were immunoprecipitated using protein A–Sepharose beads and antibodies against the target proteins for 16 hours at 4°C. Beads were washed extensively with buffer A/1% Triton X-100 and prepared for western blot analysis. For pull-down assays, extracts were incubated with 20 µg of the appropriate GST-fusion protein coupled with glutathione Sepharose 4B (Bio-World, Dublin, OH) for 16 hours at 4°C. Beads were washed extensively in the same buffer and prepared for western blot analysis. In an effort to normalize the quantity of GST-fusion proteins used in each assay, purified beads were run on a 10% SDS-PAGE along with a standard curve ranging from 1 to 20 µg of BSA. The gel was stained with Coomassie and densitometry analysis allowed us to determine the approximate volume of beads needed to obtain the desired amount of GST-fusion. Due to heavy degradation, the ful-length GST-Itch fusion was particularly hard to estimate since the binding region might be present on several bands visible by Coomassie, Ponceau and anti-GST staining. We therefore considered only the highest molecular weight band intensity in our calculations, which is likely an overestimate.

### Western blot analysis

Protein extracts and purified proteins obtained by immunoprecipitation or pull-down assays were separated by SDS-PAGE on 5–16% polyacrylamide gels. Proteins were then transferred to nitrocellulose for blotting with the appropriate primary and secondary antibodies. 0.1 µg/ml of goat anti-rabbit-HRP conjugated antibody or goat anti-mouse-HRP conjugated IgG were used (Jackson ImmunoResearch Laboratories, West Grove, PA). Antibody incubation and membrane washing were performed in PBS supplemented with 5% dry milk and 0.05% Tween 20. Immunoreactivity was detected by chemiluminescence using West-Pico SuperSignal (Thermo Fisher Scientific, Ottawa, ON).

### Quantification of pull-down assays

To quantitatively compare the interaction between Itch and the different LITAF mutants, we used 20 µg of purified GST-Itch-WW coupled to glutathione Sepharose 4B, incubated with 1 mL of protein extracts obtained from HEK-293T cells transfected with GFP-LITAF WT, GFP-LITAF Y23A, GFP-LITAF Y61A or GFP-LITAF Y23,61A. The relative binding of LITAF to Itch is obtained by measuring the ratio of the densitometry of the GFP proteins bound to beads as compared to the signal obtained with 1/10 volume of total protein extract. The densitometry measurements were performed in Photoshop CS5 (Adobe Systems Incorporated, San Jose, CA). For statistical comparison, one-way analysis of variance followed by Tukey's test was employed using SPSS software (SPSS Inc., Chicago, IL). *p*-values smaller than 0.05 were considered to be statistically significant. Data are represented as mean ± s.e.m.

### BRET experiments

For BRET analysis, HEK-293T cells (2×10^6^) were co-transfected with cDNAs coding for rLuc–Itch and different GFP fusion proteins. Forty hours post-transfection, the cells were washed in PBS, collected in 1 mL Tyrode's solution, and then diluted to 10^6^ cells/mL). Coelenterazine (Biotium, Hayward, CA, USA) was added at a final concentration of 5 µM. Total fluorescence was measured in a FlexStation apparatus (Molecular Devices, Sunnyvale, CA, USA). Luminescence and fluorescence were quantitated with a Mithras LB 940 apparatus (Berthold Technologies, Oak Ridge, TN, USA). Three measures were obtained: first, light emitted at 485±20 nm by rLuc; second, emission of fluorescence at 530±25 nm with excitation due to energy transfer from rLuc to GFP; third, emission fluorescence at 530 nm after excitation at 485 nm to measure total expression of GFP fusion proteins. The BRET ratio was defined as [(emission at 510–590 nm) – (emission at 440–500 nm)×Cf]/(emission at 440–500 nm), where Cf corresponds to (emission at 510–590 nm)/(emission at 440–500 nm) for rLuc-fused Itch expressed alone in the same experiments [34].

### Immunofluorescence analysis

Approximately 16–24 hours post-transfection cells were fixed for ten minutes in 3.7% paraformaldehyde in PBS. Cells were washed several times in PBS and were permeabilized in a 0.1% Triton X-100 in PBS solution for four minutes. Following several washes in PBS, cells were blocked for two hours at room temperature in block buffer (5% BSA (w/v), 50 mM Tris HCl (pH 7.4), 150 mM NaCl, 0.5% NP-40 (v/v). Cells were then washed several times with wash buffer (1% BSA (w/v), 50 mM Tris HCl (pH 7.4), 150 mM NaCl, 0.5% NP-40 (v/v)) and were incubated for one hour at room temperature with primary antibody diluted in wash buffer. Cells were washed several times in wash buffer and incubated for one hour at room temperature in darkness with secondary antibody diluted in wash buffer. Following several more washes in wash buffer, fluorescence was detected using a Leica DM SP2 confocal microscope (Leica, Wetzlar, Germany) and images were assembled using Adobe Photoshop CS4 (Adobe, San Jose, CA).

## Supporting Information

Figure S1 LITAF-WT alters endogenous Itch localization in different cell lines. GFP-LITAF-WT or GFP-LITAF-Y23,61A (green) was transiently transfected in Cos-7 (A), HEK-293T (B) or PAE (C) cells. After lysotracker uptake (blue), cells were fixed and immunofluorescence performed to visualize Itch protein (red).
(TIF)
