# Supplementary figures and images for: SIMPLE/LITAF Expression Induces the Translocation of the Ubiquitin Ligase Itch towards the Lysosomal Compartments
Source: PLoS One. 2011 Feb 4;6(2):e16873. doi: 10.1371/journal.pone.0016873 (PMC3033906; doi:10.1371/journal.pone.0016873)

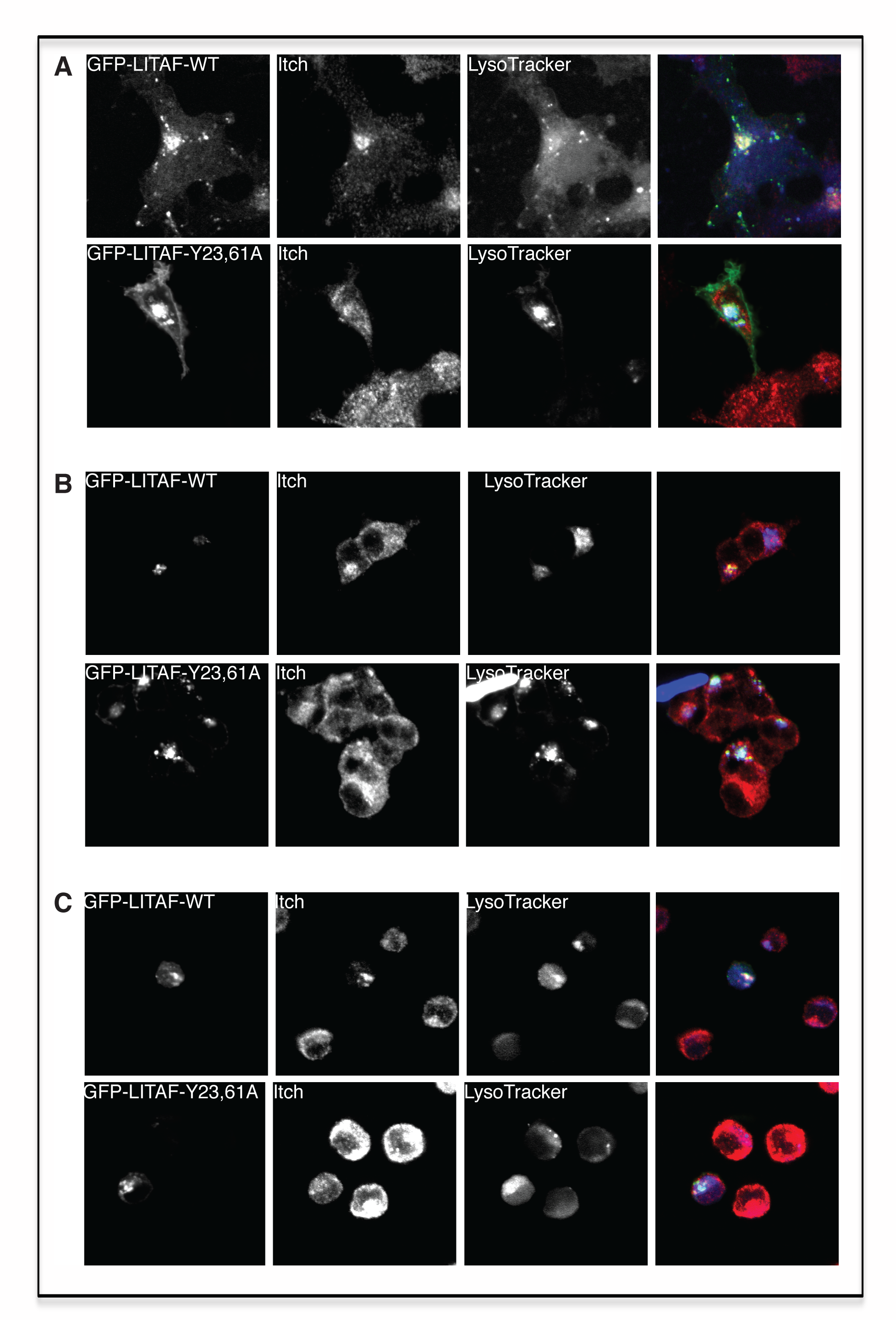

Supplement: Figure S1 — LITAF-WT alters endogenous Itch localization in different cell lines. GFP-LITAF-WT or GFP-LITAF-Y23,61A (green) was transiently transfected in Cos-7 (A), HEK-293T (B) or PAE (C) cells. After lysotracker uptake (blue), cells were fixed and immunofluorescence performed to visualize Itch protein (red). (TIF) [file pone.0016873.s001.tif]
